# Uncommon Surgical Emergencies in the Adult Gynecologic Patient: Two Cases of Missed Diagnosis of Outflow Tract Obstruction from Congenital Uterine Anomalies

**DOI:** 10.1155/2022/3179656

**Published:** 2022-11-16

**Authors:** Bailey McGuinness, Natalia Llarena, Tommaso Falcone, Elliott G. Richards

**Affiliations:** ^1^Cleveland Clinic, Cleveland, USA; ^2^Cleveland Clinic, London, UK

## Abstract

Gynecologic emergencies may result from congenital uterine anomalies (CUAs) with outflow tract obstruction. Not limited to the “classic” presentation of an adolescent amenorrheic pain patient, such anomalies should be part of the differential diagnosis for adult female patients presenting with severe pelvic pain. Obstructed rudimentary noncommunicating cavitary horns may result in severe chronic or acute pain and necessitate urgent surgical management. While two-dimensional (2D) ultrasound is often the initial diagnostic tool, three-dimensional (3D) ultrasound and MRI can accurately delineate CUAs for definitive diagnosis. When excision of a rudimentary horn is required, a laparoscopic approach is preferable. This case series focuses on two adult patients with severe pelvic pain due to unicornuate uteruses with obstructed noncommunicating cavitated rudimentary horns. Both cases involve a delayed diagnosis, the inability to make the diagnosis at standard surgical observation, and the resultant need for urgent surgical management.

## 1. Background

Gynecologic emergencies are conditions that threaten a woman's life, sexual function, or fertility [1]. Gynecological emergencies are relatively common and can be categorized as acute pain (e.g., ectopic pregnancy, adnexal torsion, hemorrhagic ovarian cyst, and outflow tract obstruction), gynecologic hemorrhage (e.g. vulvovaginal trauma, abnormal uterine bleeding, abortion, malignancy, and gestational trophoblastic disease), and infection or sepsis (pelvic inflammatory disease, tubo-ovarian abscess, retained foreign object, and septic abortion).

Gynecologic emergencies related to CUAs typically result from either ectopic pregnancy or outflow tract obstruction. However, outflow tract obstruction is not generally included in the differential diagnosis of acute pelvic pain in adults, as the classic presentation is an adolescent with primary amenorrhea and cyclic abdominal pain. However, CUAs may present with acute abdominal pain in adults in the setting of outflow tract obstruction.

While patients with CUAs may present with pelvic pain, abnormal uterine bleeding, or genital tract infection, many patients with CUAs are asymptomatic, and diagnosis often occurs during physical exam or as an incidental finding on imaging study. On physical exam, patients may have a double or no cervix, vaginal septum, or a shortened or absent vagina. Incidental diagnosis is often made at the time of pelvic ultrasound, magnetic resonance imaging (MRI), CT scan, or hysterosalpingogram. The accepted imaging standard for Müllerian anomalies is 3D ultrasound.

A unicornuate uterus includes a single normally developed cervix, uterus, and fallopian tube. The Müllerian duct that failed to develop properly may be completely absent or a rudimentary horn may exist. The frequency of unicornuate uterus is 10% of all CUAs [2]. Unicornuate uterus is rare, occurring in 1/1,000 to 1/5,400 women [3]. The majority of women (74-90%) with unicornuate uteruses have rudimentary horns [3]. In 78% of patients, presentation occurs after age 20, with a mean age of 23-26 [4].

There is no universally accepted classification system for CUAs. The European Society of Human Reproduction and Embryology (ESHRE) and the European Society of Gynecological Endoscopy (ESGE) [5], the American Society of Reproductive Medicine [6], Acien's classification [7], Congenital Uterine Malformation by Experts [8], and others have proposed classification systems. The most widely accepted is the ESHRE/ESGE consensus on the classification of female genital tract congenital anomalies [5].

Rudimentary horns can be described as follows: (1) horns with or without an endometrial cavity; (2) horns with or without a communicating tract between the cavities of the rudimentary horn and unicornuate uterus; (3) horns fused or separate from the unicornuate uterus [9]. Cavitary noncommunicating rudimentary horns pose the greatest clinical threat, as they most commonly result in pelvic pain and dysmenorrhea due to hematometra or endometriosis [9]. Outflow tract obstruction may occur via a nonfunctional or torsed fallopian tube, salpingectomy or tubal ligation procedures, hydrosalpinx, malignancy, or compression from a pelvic mass, cyst, or fibroid.

Although there are several published case reports of the management of unicornuate uteruses with rudimentary horn [10, 11, 12, 13], there are no reported cases to our knowledge of *obstructed* noncommunicating cavitated rudimentary horns resulting in urgent surgical management in the adult female patient.

**Figure 1:**
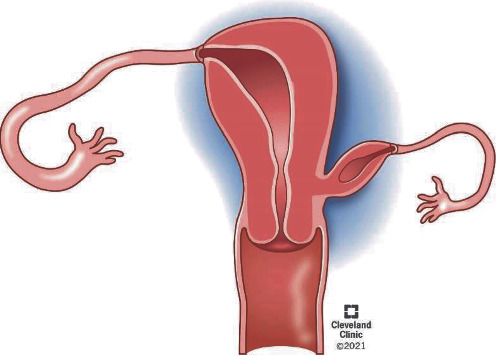
Unicornuate uterus with noncommunicating cavitary rudimentary horn.

Here, we present two adult patients, both with pain due to unicornuate uteruses with obstructed noncommunicating cavitated rudimentary horns, ESHRE/ESGE class U4aC0 (Figure 1) [5]. Both cases involve a delayed diagnosis in the setting of severe, intractable pelvic pain. The most critical aspect of emergency surgical management in cases of adult-onset CUA is the clear identification of the relationship of the rudimentary horn to the uterus through appropriate preoperative imaging.

## 2. Case Presentation #1

A 29-year-old gravida one para one female with essential hypertension and a body mass index (BMI) of 60.7 kg/m^2^ was evaluated in the out-patient setting for an acute worsening of chronic pelvic pain at the Cleveland Clinic Fertility Center in Beachwood, Ohio. At the age of sixteen, she began to experience pelvic pain that was managed intermittently with menstrual suppression via medroxyprogesterone acetate injections.

Nine years prior to her presentation, the patient conceived a child and had an early term cesarean delivery for oligohydramnios, intrauterine growth restriction (IUGR), and breech presentation. The operative report noted an 8 cm right fundal fibroid with no uterine abnormalities noted. Five years prior to her presentation, she had a laparoscopic left ovarian cystectomy for a mature teratoma; that operative report noted a 5 cm mass coming off the right fundal aspect of the uterus with “chocolate” fluid contents. Despite this finding, no definite treatment was undertaken, and the patient continued to have chronic pelvic pain.

Six months prior to presentation, the patient stopped treatment with medroxyprogesterone acetate and began to experience intractable pain despite multiple regimens of NSAIDs and narcotics. In the month prior to evaluation in our clinic, she had an acute worsening of pelvic pain, which resulted in five emergency department (ED) visits and two in-patient admissions for pain control. During this time, she was noted to have leukocytosis with a left shift.

**Figure 2:**
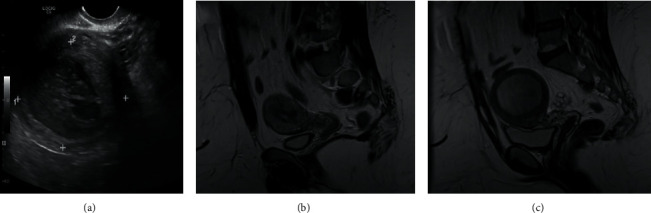
(a) 2D scan read as fibroid with central necrosis. (b, c) Communicating and noncommunicating horns on MRI.

During these ED encounters, the rudimentary horn was read as a fibroid with central necrosis on ultrasound (Figure 2(a)), and anatomy was reported to not be well visualized on CT scan. Ultimately, with her pain continuing to persist, her general gynecologist ordered an outpatient MRI, which showed a unicornuate uterus with an 8 cm noncommunicating rudimentary right horn (Figures 2(b) and 2(c)). The patient was referred to our Reproductive Endocrinology and Infertility practice. Upon presentation to our clinic and shortly after yet another ED visit for severe pain, she was rapidly evaluated and consented for urgent fertility-sparing surgical management of the rudimentary horn via laparoscopic hemi-hysterectomy. Due to the patient's extreme obesity, the decision was made to proceed with robotic-assistance using the Xi da Vinci Robot (See Supplemental Video) (available here).

Following induction of anesthesia and abdominal entry, the robot was side-docked to allow easy access to the vagina, with camera port at the umbilicus, three additional robotic ports in left and right lower quadrants, and an assistant port in right lower quadrant.

**Figure 3:**
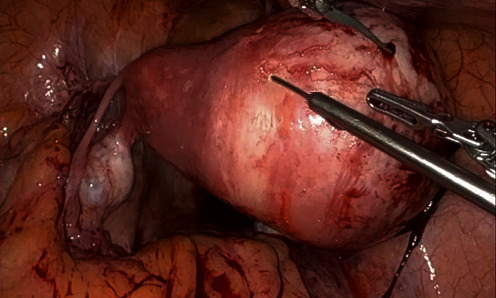
Intraoperative view of distended noncommunicating horn.

The procedure began with a survey of the pelvis for endometriosis, which revealed no macroscopic evidence of endometriosis. The rudimentary horn's fallopian tube was attenuated and did not appear to be a functional outlet for the horn. The rudimentary horn was grossly distended with blood to approximately 9 cm (Figure 3). The bilateral ovaries and contralateral fallopian tube were present and normal appearing.

The hemi-hysterectomy of the rudimentary horn was performed incorporating aspects of both laparoscopic hysterectomy and laparoscopic myomectomy. The surgery began with identification of the ipsilateral ureter, opening of the broad ligament, and use of the da Vinci vessel-sealer to coagulate and transect the round ligament, the utero-ovarian ligament, and the uterine vessels, particularly the ascending branch of the uterine artery. A laparoscopic tenaculum in the third robotic arm was used for retraction and manipulation of the rudimentary horn. Adhesions of the bladder to the anterior aspect of the rudimentary horn were taken down, and the area of fusion between the rudimentary horn and unicornuate uterus was injected with dilute vasopressin to minimize blood loss during incision into the myometrium. A plane was developed between the unicornuate uterus and the rudimentary horn, and the rudimentary horn was removed along with the ipsilateral fallopian tube. The excised horn was moved out of the field to allow for visualization and closure of the myometrial defect using a 0-barbed suture in three layers. The umbilical incision was extended to 4 cm, and the excised horn and right fallopian tube were placed in a specimen bag and removed from the abdomen. At the conclusion of the procedure, the pelvis was irrigated, and all pedicles were hemostatic. Pathology was consistent with benign uterine tissue, and there was no evidence of microscopic endometriosis.

At a follow-up appointment, four months after surgery, the patient's pelvic pain had resolved. However, the patient reported ongoing struggle with a dependence on opioids prescribed during the months-long episode of severe pain prior to her diagnosis and treatment.

## 3. Case Presentation #2

A 40-year-old nulligravida with BMI 36 kg/m^2^ was evaluated in the out-patient setting for infertility. Her care was transferred to at the Cleveland Clinic Fertility Center in Beachwood, Ohio from another IVF center, where she had undergone one in vitro fertilization (IVF) cycle, notable for an inaccessible left ovary at oocyte retrieval and no resulting embryo viable for transfer.

The patient's medical history was notable for vulvodynia (with inability to have coitus), endometriosis, obstructive sleep apnea, hypertension, and anxiety. Due to her vulvodynia, the patient was unable to tolerate vaginal exams or procedures without anesthesia. Hence, two attempts at hysterosalpingo-contrast sonography failed. She was diagnosed with a “bicornuate uterus” via transabdominal ultrasound. In addition to making this diagnosis, the previous IVF center performed a laparoscopic left salpingectomy for hydrosalpinx, excision of endometriosis, and left oophoropexy to improve access to the left ovary for future oocyte retrieval procedures.

**Figure 4:**
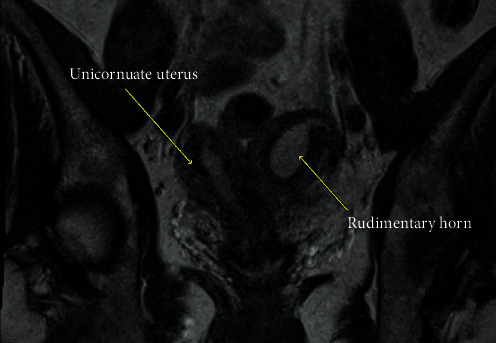
MRI of communicating and noncommunicating horns.

Upon transfer of care to our institution, the patient underwent a second cycle of IVF, resulting in successful oocyte retrieval from both ovaries and one resultant euploid embryo, with evidence of an endometrial polyp seen during egg retrieval. Hysteroscopic polypectomy was performed in preparation for embryo transfer. During exam under anesthesia, a hypoplastic upper portion of the vagina was noted. Only a single cervix, uterine cavity, and tubal ostium were noted. No tract to a second cavity was identified, regardless of multiple surveys of the lower uterine segment and cervical canal, which was inconsistent with her diagnosis of bicornuate uterus. An MRI with contrast was ordered for definitive diagnosis (Figure 4). The MRI showed a right unicornuate uterus with a noncommunicating cavitary left rudimentary horn that was dysmorphic and moderately distended.

It was presumed that the patient's recent left salpingectomy had resulted in outlet obstruction of the rudimentary horn. She was scheduled for a robotic-assisted laparoscopic hemi-hysterectomy of the rudimentary uterine horn, which was scheduled six weeks later as an elective procedure given the absence of any symptoms. Over the next two weeks, the patient experienced the onset of severe pelvic pain and presented to the emergency department twice for pain control. Due to the severity of her symptoms, she was scheduled for urgent surgical management. Due to lack of availability of the da Vinci robot, we proceeded with a conventional laparoscopic hemi-hysterectomy.

The camera port was placed at the umbilicus. A laparoscopic port was placed in the left and right lower quadrants. A 5 cm transverse minilaparotomy was made two fingerbreadths above the pubic symphysis. The GelPort laparoscopic system was used at the site of the minilaparotomy to allow swift alternate access between laparotomy and laparoscopic techniques to improve surgical efficiency and outcomes [14].

**Figure 5:**
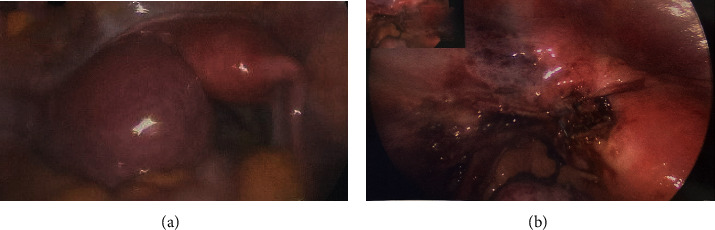
(a) Intraoperative view of distended noncommunicating left horn. (b) Shallow myometrial defect after removal of left horn.

On survey of the abdomen and pelvis, the left ovary and rudimentary horn were densely adherent to one another and the left pelvic side wall. There was no macroscopic evidence of endometriosis. The rudimentary horn was noted to be distended with blood (Figure 5(a)). The left fallopian tube was surgically absent. The right unicornuate uterus, fallopian tube, and ovary were normal appearing. Patency of the right fallopian tube was established via chromopertubation.

Careful attention was paid to lyse adhesions between the rudimentary horn, left ovary, and pelvic side wall along avascular planes to ensure minimal bleeding. Traction and countertraction were used to create tension to allow for clearer identification of avascular planes. Ureterolysis was performed in the usual fashion. The left ureter was noted to be adherent to the left ovary and rudimentary horn. Blunt dissection was used to lyse fine adhesions, while sharp dissection was used for dense adhesions, using both laparoscopic scissors and a bipolar vessel sealing (LigaSure) device.

Once adhesiolysis was complete, the steps of a hemi-hysterectomy were initiated. The connecting area between the rudimentary horn and unicornuate uterus was injected with dilute vasopressin to minimize blood loss. The hemi-hysterectomy was then performed using the laparoscopic LigaSure device to open the broad ligament and to coagulate and transect the round ligament, the utero-ovarian ligament, and the uterine vessels, as before. Adhesions of the bladder to the anterior aspect of the rudimentary horn were taken down. A plane was carefully developed between the firm attachment of the unicornuate uterus and the rudimentary horn. The rudimentary horn was then excised and moved out of the field to allow for visualization and closure of the myometrial defect. A 0-barbed suture was used to close the small and shallow myometrial defect in a single layer. The excised horn was removed via the minilaparotomy incision. Cystoscopy was performed and no injury to the bladder was noted; bilateral ureteral jets were appreciated. At the conclusion of the procedure, the pelvis was irrigated, and the entire pelvis was noted to be hemostatic (Figure 5(b)).

Pathology was consistent with endometriosis and 45.9 g of benign uterine tissue, measuring 4.7 x 4.7 x 3.7 cm. At a follow-up appointment, two weeks after surgery, the patient's pelvic pain had resolved. Table. 1.

## 4. Discussion

### 4.1. Diagnosis and Imaging

These cases demonstrate the importance and difficulties in obtaining a definitive diagnosis as well as the negative implications of perpetuating a false diagnosis. The diagnosis of unicornuate uterus can be difficult, even with direct visualization during laparotomy (Case 1) and laparoscopy (Cases 1 and 2). It is important to note that the reported prevalence of CUAs is 5.5% in an unselected population, 8% in infertile patients, 12.3% in patients with a history of miscarriage, 24.5% in patients with a history of infertility and miscarriage, and 29% in patients with renal anomalies [15, 16]. CUAs are associated with renal, skeletal, and abdominal wall anomalies. 20-30% of patients with Müllerian defects have renal abnormalities [2, 17]. Physicians should be aware of at-risk populations who may benefit from evaluation for CUAs and renal ultrasound. This includes patients with primary amenorrhea, infertility, dysmenorrhea, known renal anomalies, recurrent pregnancy loss, and obstetric complications known to be associated with CUAs, as discussed below. Due to hematometra and retrograde menstruation, there is an increased prevalence of endometriosis in patients with a rudimentary horn.

The gynecologic emergencies presented here could have been avoided if these patients received an accurate diagnosis sooner. In Case 1, the rudimentary horn was previously believed to be a subserosal degenerative fibroid. In Case 2, the rudimentary horn was believed to be a bicornuate uterine horn. Physicians should be aware that the misclassification of CUAs can be perpetuated in subsequent patient records, even between practices. Here, misdiagnoses led to severe chronic and acute pelvic pain which both culminated in a surgical emergency. Both patients had a history of prior surgical procedures; if the rudimentary horn had been previously or presently diagnosed, a hemi-hysterectomy could have been performed at that time, eliminating the need for additional surgery in an urgent setting.

2D ultrasound is the initial imaging modality for evaluation of CUAs; this is due to ultrasound's availability, relative cost efficiency, noninvasive nature, and ability to provide information about other pelvic structures and kidneys [18]. Unfortunately, conventional ultrasound has a sensitivity of only 26%, and 3D ultrasound or MRI provides improved sensitivity and better delineation of the anatomy. 3D ultrasound is considered the “gold standard” for diagnosis of CUAs. MRI should be reserved for complex situations where ultrasound findings are uncertain and a definitive diagnosis would alter patient care.

Chromopertubation is an important part of the intraoperative investigation, as it may be used to determine whether one or both uteruses communicate with the cervix. In addition, thorough examination of the vagina and cervix under anesthesia is critical to ensure that the presence of a duplicated cervix is not missed.

### 4.2. Special Considerations for Obese Patients: Robotic-Assisted or Conventional Laparoscopy

Patients with a high BMI present unique surgical challenges. These challenges often lend themselves to a robotic-assisted or conventional laparoscopic approach. For obese patients, assistance of the robot provides several advantages, such as reduced operating time in patients with a BMI ≥ 50 kg/m^2^, superior ergonomics, reduced surgeon fatigue, and improved trochar movement due to instrument wrist articulation in the setting of a thick abdominal wall [19]. Due to the urgent need for surgical management in the setting of a gynecologic emergency for outflow tract obstruction, the staff and equipment necessary for a robotic-assisted procedure may be unavailable. Therefore, it is important that the surgeon be capable of performing this surgery by conventional laparoscopy or considers referral to a specialist in minimally invasive surgery. We provide several tips for performing this surgery in the Supplemental Video (available here).

### 4.3. Considerations for Future Conception

For patients desiring fertility, the decision to attempt conception immediately versus waiting should depend on the extent of the defect and repair of the myometrial bed. For patients with a separate rudimentary horn from the unicornuate uterus, the patient may attempt conception once they are fully recovered from surgery. For patients with a fused rudimentary horn, the decision to delay conception should be individualized. For extensive myometrial defects and repair, we recommend following similar guidelines as patients who undergo myomectomy (e.g., delaying conception for three months and counseling regarding the timing and mode of delivery).

Patients should be counseled regarding the obstetric risks in the setting of a unicornuate uterus. CUAs can lead to a multitude of obstetric complications including spontaneous abortion and recurrent miscarriage, preterm rupture of membranes and birth, abnormal placentation, IUGR, cervical insufficiency, fetal malpresentation, hypertension disorders, cesarean birth, bleeding, and rupture of a rudimentary horn. The patient in Case 1 is an excellent example of obstetric complications in the setting of unicornuate uterus: placental insufficiency, IUGR, fetal breech presentation, and cesarean birth.

## 5. Conclusion

Obstructed rudimentary noncommunicating cavitary horns may result in severe chronic or acute pain and necessitate urgent surgical management. Timely diagnosis is critical. Outflow tract obstruction of a CUA should be part of the differential diagnosis for adults presenting with pelvic pain. 3D ultrasound and MRI can accurately diagnose CUAs. Nondefinitive imaging and/or the inability to make the diagnosis at standard surgical observation may delay the diagnosis of a CUA and can eventually require urgent surgical management. When excision of a rudimentary horn is required, it should be removed laparoscopically. A robotic-assisted approach facilitates minimally invasive surgery in patients with a high BMI.

## Figures and Tables

**Table 1:** Patient characteristics.

	Case #1	Case #2
Age	29 years	40 years
Gravidity parity	G1P1	G0P0
BMI	60.7 kg/m^2^	36 kg/m^2^
Prior surgery	(i) Cesarean section (ii) Laparoscopic ovarian cystectomy	(i) Laparoscopic unilateral salpingectomy (ii) Hysteroscopy
Mullerian anomaly	Unicornuate uterus	Unicornuate uterus
ESHRE/ESGE classification [5]	U4aC0V0	U4aC0V4 (Hypoplastic upper vagina)
Description of rudimentary horn	Obstructed Noncommunicating Cavitated	Obstructed Noncommunicating Cavitated
Etiology of rudimentary horn outflow tract obstruction	Nonfunctional fallopian tube	Salpingectomy
Approach to hemi-hysterectomy	Robotic-assisted laparoscopy	Laparoscopy

## Supplementary Materials

Supplementary Materials Supplemental video: https://drive.google.com/file/d/1L4olRHN98WAnxKByz9xaWytghu-3ePC3/view
