# Supplementary material for: Uncommon Surgical Emergencies in the Adult Gynecologic Patient: Two Cases of Missed Diagnosis of Outflow Tract Obstruction from Congenital Uterine Anomalies
Source: Case Rep Obstet Gynecol. 2022 Nov 16;2022:3179656. doi: 10.1155/2022/3179656 (PMC9683970; doi:10.1155/2022/3179656)
Supplement: Supplementary Materials — Supplemental video: https://drive.google.com/file/d/1L4olRHN98WAnxKByz9xaWytghu-3ePC3/view [file 3179656.f1.docx]

SUPPLEMENTAL VIDEO

https://drive.google.com/file/d/1L4olRHN98WAnxKByz9xaWytghu-3ePC3/view
